# Combined assessment of lysine and *N*-acetyl cadaverine levels assist as a potential biomarker of the smoker periodontitis

**DOI:** 10.1007/s00726-024-03396-4

**Published:** 2024-06-08

**Authors:** Md Haroon Rashid, Sandhya Pavan Kumar Yellarthi, Pavan Kumar Yellarthi, Brinda Goda Lakshmi Didugu, Anitha Mamillapalli

**Affiliations:** 1 Department of Biotechnology, School of Science, GITAM (Deemed to be University), Visakhapatnam, Andhra Pradesh 530 045 India; 2 Department of Periodontics and Oral Implantology, GITAM Dental College and Hospital, Visakhapatnam, Andhra Pradesh 530 045 India; 3 Department of Oral Medicine and Radiology, GITAM Dental College and Hospital, Visakhapatnam, Andhra Pradesh 530 045 India

**Keywords:** Periodontitis, Smokers, Reverse smokers, Polyamines, N1,N8-diacetyl spermidine, *N*-acetyl cadaverine

## Abstract

Periodontitis is an inflammatory condition of supporting structures of teeth leading to attachment and bone loss. Cigarette smoking is the single most important and modifiable risk factor with 5 to 20-fold susceptibility for periodontal diseases. Reverse smoking is a peculiar habit of smoking where the lit end is kept inside the mouth, which is predominant in the northern coastal districts of Andhra Pradesh. Polyamines are biologically active amines involved in tissue regeneration and modulation of inflammation. The study aimed to evaluate polyamines and check their utility as a marker in detection of periodontitis among different groups. Total polyamine levels showed significant increase in reverse smokers with periodontitis when compared to the other groups. Qualitative analysis by thin layer chromatography showed three polyamine bands with varying intensity among the different groups. Mass spectrometric and NMR analyses of the three bands identified them as N1, N8-diacetyl spermidine, *N*-acetyl cadaverine and lysine. Most significantly elevated levels of lysine was observed in the smoker and reverse smoker periodontitis groups when compared to healthy and non-smoker periodontitis groups. The significantly elevated levels of *N*-acetyl cadaverine could be responsible for the more destruction of periodontium in the reverse smoker group. Antioxidant potential decreased significantly in different smoker periodontitis groups. The present study suggests that the quantitative analysis of salivary polyamines, lysine and *N*-acetyl cadaverine can aid as an easy noninvasive diagnostic method for assessing the periodontal status, especially in smokers.

**Supplementary Information:**

The online version contains supplementary material available at 10.1007/s00726-024-03396-4.

## Introduction

Periodontal disorders are a group of chronic inflammatory condition of the tooth's supporting structures that results in attachment and bone loss, characterized by gingival inflammation, clinical attachment loss, periodontal pockets, gingival recession, alveolar bone loss, and eventually tooth loss (Cortelli et al. 2021). Periodontitis is a known risk factor for a variety of systemic diseases including diabetes, cardiac diseases, adverse pregnancy outcomes, metabolic syndrome, and rheumatoid arthritis, as well as a reduction in quality of life. As a result, identifying and diagnosing active periodontal disease is critical (D’Aiuto et al. 2008; De Smit et al. 2015; Sanz et al. 2020).

According to the World Health Organization (WHO), smoking is a threat to public health; it kills over eight million individuals each year, with nearly seven million deaths caused by direct smoking and approximately 1.2 million fatalities resulting from indirect exposure to smoke (who fact sheet 2022*).* The reverse practice of smoking is most common in the southern state of Andhra Pradesh Srikakulam district. In a residence-to-residence survey of 10,169 villagers with precancerous and cancerous oral conditions, 43.8% had been reverse smokers, with respect to female: male proportion of 1.7: 1 (Pindborg et al. 1971).

Cigarette smoking is a particularly important and well-established risk factor for periodontal disease growth and development (Haber et al. 1993; Kolte et al. 2012). Saliva is an intricate biological fluid made up of several distinct substances. Saliva is quickly gaining popularity as a diagnostic tool due to its benefits such as low cost of acquisition, availability of multiple sampling, ease of access, and avoidance of biopsies. Saliva biochemical analysis aids in predicting the risk of disease onset and degree of severity, monitoring the advancement of the disease, and assessing therapeutic efficacy in oral diseases (Giannobile et al. 2009). Diagnosing and identifying active periods of periodontal disease in patients at risk for tissue breakdown remains challenging for the clinician.

Polyamines (PAs) are low-molecular-weight aliphatic organic compounds that contain two or more amino groups and are found in significant amounts in almost all prokaryotic and eukaryotic cell types (Mustafavi et al. 2018). High intracellular PAs are known to be involved in many cellular processes, cell growth and proliferation, chromatin architecture modulation, stabilization of DNA and gene transcription as well as translation (Henderson Pozzi et al. 2009; Igarashi and Kashiwagi 2018). Several studies have shown that spermidine (Spd) and spermine (Spm) are involved in the prevention of bone loss in ovariectomy mice (Yamamoto et al. 2012). The involvement of PAs in cell migration was found to be through PA regulator *AMD1 *(Lim et al. 2018). Putrescine (Put) was discovered to play an important role in the periodontitis recovery period (Ishida et al. 1983). Lysine is an amino acid essential for integrity and dentally attached epithelial cell renewal. The bacteria deplete lysine, depriving host-attached cells of this essential amino acid nutrient, destroying the bacterial barrier, and causing inflammation and periodontitis. Salivary cadaverine and its derivative, *N*-acetyl cadavrine were higher in periodontal inflamed surface area which lead to enhanced gingival cervical fluid (GCF). This helped in slow destruction of the periodontium (Goldberg et al. 1994; Levine and Lohinai 2021;Sakanaka et al. 2017). As the periodontitis disease characteristics match with the multiple roles played by PAs, this study aimed to analyze and check for differences in PA levels among periodontally and systemically healthy, non-smokers, smokers, and reverse smokers with periodontitis.

## Materials and methods

### Study design

A total, of 80 individuals satisfying the inclusion and exclusion criteria from the outpatient department, GITAM Dental College & Hospital, Visakhapatnam, India, who consented to 13 ml of saliva sampling were included in the study. 20 systemically and periodontally healthy individuals, 20 non-smokers with periodontitis (P + NS), 20 smokers with periodontitis (P + S), 20 reverse smokers with periodontitis (P + RS), formed the study groups. Male/Female individuals aged 35–60 years, systemically and periodontally healthy individuals, non-smokers with periodontitis, smokers, and reverse smokers with periodontitis who have smoked ≥ 10 cigarettes per day for a minimum of 5 years were included in the study. Pregnant patients, with teeth showing trauma from occlusion, and patients with a history of drug intake for any systemic diseases were excluded from the study. The study was explained in local language and written informed consent was obtained from the participants. Ethical clearance of the study was taken from the GITAM Dental college and Hospital ethical committee, Vishakhapatnam (AP), India.

### Case definition for periodontitis

A periodontitis case definition has been done according to Papapanou et al.(2018).

- Interdental clinical attachment loss (CAL) of > 2 mm, when the Cemento Enamel junction (CEJ) is detectable at two or more non-adjacent teeth.
- Buccal or oral CAL of at least 3 mm with pocketing > 3 mm.
- CAL cannot be associated with non-periodontal cases.
- Presence of Radiographic bone loss.
- Bleeding on probing in sites measuring > 4 mm.

### Case definition for smokers and reverse smokers

Reverse smoking: The lit end of a hand-rolled tobacco leaf is kept in the mouth instead of the unburnt end.

Smoked ≥ 10 cigarettes per day for a minimum of 5 years (Michael et al. 2007).

### Collection of salivary samples

Whole saliva samples (unstimulated) were collected in a sterile conical tube from each patient before clinical evaluation according to the established protocol by passive drool or spit technique after the mouth was rinsed with water (Bachtiar et al. 2021). Patients were instructed not to consume any type of food or use mouthwash for at least 1 h before saliva collection. Around 10 ml saliva was collected in 15 ml sterile plastic centrifuge tube provided by Tarson Pvt. Ltd. Collected saliva samples were centrifuged at 1500 rpm for 30 min at 4 °C and the resulting supernatant was separated in a 2 ml sterile centrifuge tube and stored at − 80 °C until further analysis. Quantification of PAs in saliva samples was analysed by derivatization, fluorescent labelling and estimation by fluorescence spectrophotometer.

### Dansylation of PA

Total PAs was estimated according to a published protocol with some modifications (Lima et al. 2023). 100 µl of centrifuged saliva sample was used to estimate PA.

### Preparation of PA standards

Spd, Spm, and Put standard PAs were prepared in distilled water at a concentration of 1 mg/ml. 20 µl of each PA standard was processed for dansyl chloride labelling. The labelled standard sample was used as marker and was separated in TLC along with the labelled PAs of saliva samples.

### Estimation of PA

PA quantification was carried out following the extraction of dansylated PAs into the organic phase. A fluorescence spectrometer was used to measure PAs (Lima et al. 2023). Spd was used as a control, and the results were expressed as micrograms of PAs present in 200 µl of saliva. The 350 nm and 495 nm were used for excitation and emission respectively. A total of 20 samples (*n* = 20) were analyzed in each group.

### TLC analysis

TLC was used to separate the extracted dansylated PAs from the saliva sample according to an earlier published protocol (Lima et al. 2023). A pre-coated silica gel TLC aluminium sheet (60 F254 Merck Millipore) (20 × 10 cm) was used. 150 µl of the extracted PAs were loaded onto the TLC plate at the loading point. A glass chromatographic tank (2515 cm) was used for linear ascending development and was pre-saturated for 30 min with ethyl acetate and *n*-hexane (3:4, v/v). The separated bands of dansylated PAs were observed, in gel documentation system using UV light and densitometric analyses were performed of the separated bands using Bio-Rad Image Lab 6.0.

### Mass spectrometry analysis of PA

Distinct bands from the TLC plate were selected, pooled, and processed with a slight modification. PAs bands were cut from silica plates with a sharp blade and stored in 1.5 ml tubes. The PAs in the saliva sample were measured using a Waters Xevo G2-S Q-TOF mass spectrometer as described earlier (Lima et al. 2023).

### NMR analysis of PA

The number and nature of hydrogen atoms and carbon atoms in PAs eluted from TLC were determined using 1H NMR and 13C NMR analysis. The 1H spectra of PAs were obtained using an NMR spectrometer set to 400 MHz, chloroform-d as the solvent, and tetramethylsilane (TMS) as the internal standard.

### Analysis of antioxidant potential of the salivary samples

The antioxidant potential of the saliva samples of all the groups was analyzed using 1,1-diphenyl-2-picrylhydrazylc (DPPH), (Brand-Williams et al. 1995) and superoxide dismutase (SOD) (Beauchamp and Fridovich 1971) assays. 100 µl of saliva was used for each assay with twenty samples in each group (*n* = 20).

### Statistical analysis

The one-way ANOVA was used to investigate differences in mean PA levels, the DPPH and SOD levels between groups. The TUKEY Post HOC test was used to identify means that are significantly different from one another; all tests were used depending on the nature of the distribution. To determine screening ability, the receiver operating characteristic curve (ROC) curve was drawn, and *p* < 0.005 was considered as statistically significant. Data were analysed using SPSS version 25.0 (IBM Corporation, USA).

## Results

### Quantitative changes in salivary PA levels

**Fig. 1:**
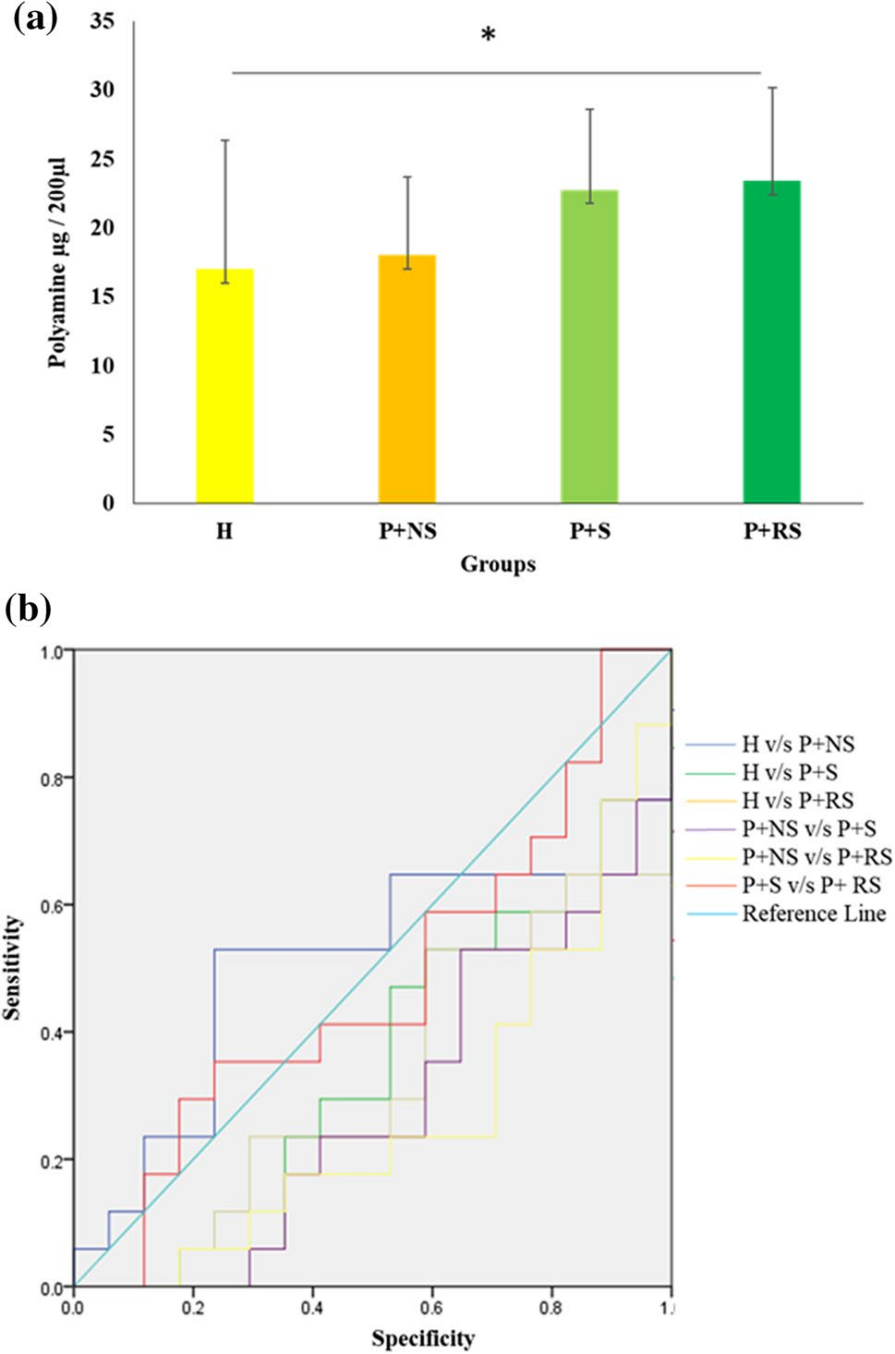
Quantitative analysis of PAs in saliva samples. **a** Total polyamines were determined in H, P + NS, S + P and P + RS; the X-axis indicates the study group, Y-axis indicates the measurement of PAs. *Refers to the significant difference between healthy and RSP (*p* < 0.05). **b** Receiver operating characteristic (ROC) curve for screening ability of PA

Total PA levels were estimated in the salivary samples collected from 80 individuals (20 each in P + RS, P + S, P + NS, and systemically and periodontally healthy individuals) who were in the age group of 35–60 years. In the present study, an inter-group comparison of PAs demonstrated a statistically significant difference (*p* < 0.05) with the highest levels of PAs in reverse smokers with periodontitis (23.43 ± 6.73) followed by smokers with periodontitis (22.78 ± 5.87), non-smokers with periodontitis(18.02 ± 5.65), and healthy individuals (17.04 ± 9.31). (Table S1, Fig. 1a).

The pairwise comparison demonstrated that the P + RS group had significantly (*p* < 0.05) higher levels of PAs compared to healthy individuals. PA levels were elevated in all the periodontitis groups compared to healthy individuals (Table S2, Table S3).

Receiver operating characteristic curve was utilized to calculate the screening ability of PAs for periodontitis. It was observed with reference to healthy individuals, PAs had a sensitivity of 52.9% and specificity of 58.8% in non-smokers with periodontitis, while in smokers with periodontitis the sensitivity was 58.8% and specificity was 52.9% and in reverse smokers with periodontitis the sensitivity was 64.7% and specificity was 52.9% for the detection of periodontitis (Fig. 1b, Table S4).

### Qualitative differences in salivary PAs among different groups

**Fig. 2:**
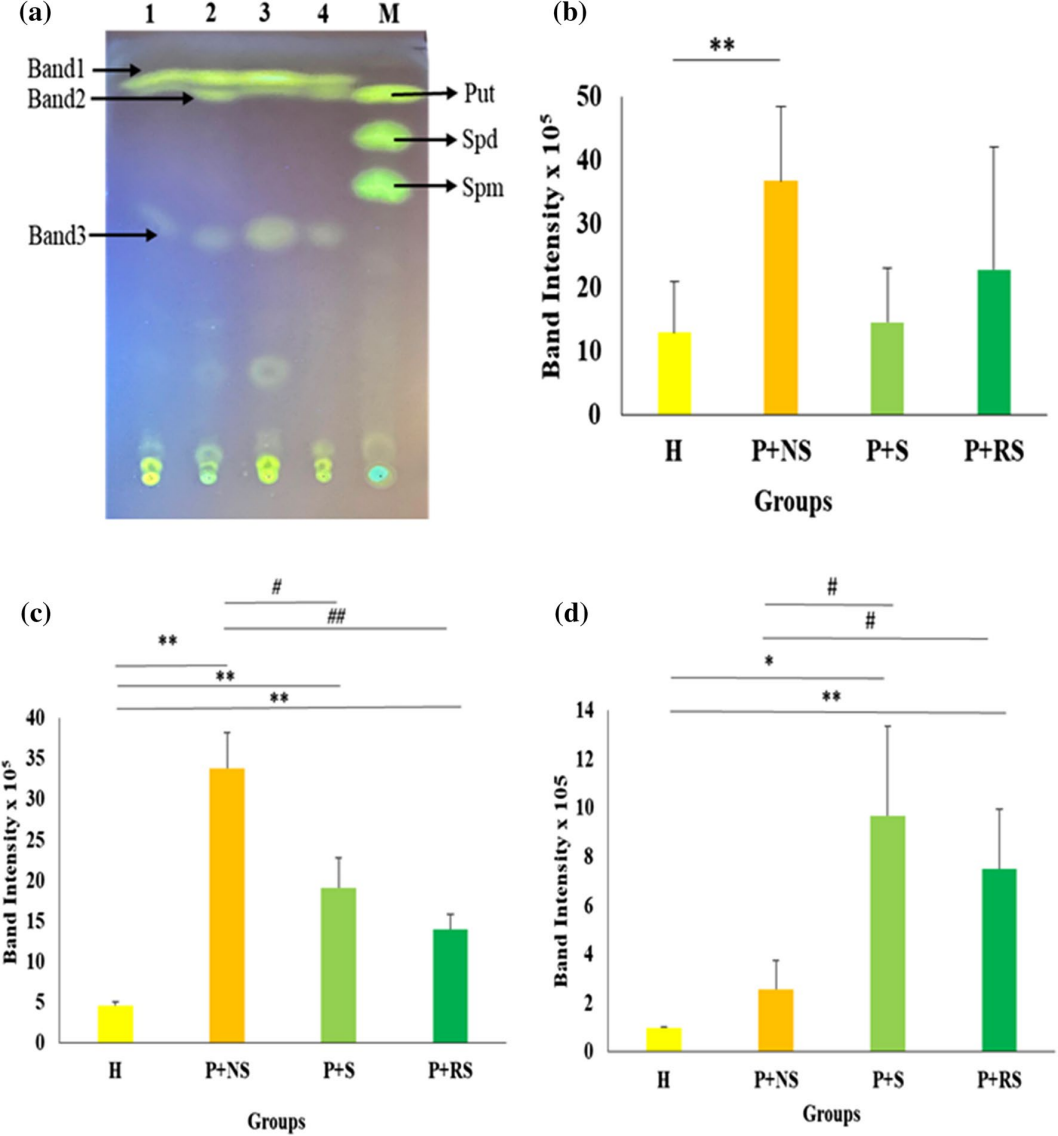
Separation and estimation of dansylated PAs by silica TLC. **a** Separated PAs on TLC. 1, 2, 3, 4 represent H, P + NS, S + P and P + RS respectively. **M** denotes standard PAs. **b**, **c**, and **d** Densitometric analysis of PA band 1, band 2, and band 3 respectively. * and ** indicates *p* < 0.05 and *p* < 0.01 between H and other groups. # and ## indicates *p* < 0.05 and *p* < 0.01 between non-smoker and smoker groups (*n* = 3)

Further, TLC analysis was carried out to understand the qualitative differences in salivary PAs extracted from different groups. TLC fractionation showed three distinct PA bands in all the groups (Fig. 2A). The bands corresponding to standard Spd and Spm were not observed in the healthy and different periodontitis groups. A band similar in mobility to Put was observed in healthy and periodontitis groups which was analyzed further. An inter-group comparison of of band 1, band 2 and 3 intensities showed statistically significant difference (*p* < 0.05 & *n* = 3) (Table S5).

The pairwise comparison of band 1 intensity revealed that it is highly significantly increased in P + NS group when compared to healthy group. P + S and P + RS groups did not show any significant change in intensity when compared to healthy group. Comparision of band 1 intensity between smoker and reverse-smoker groups also did not show any significant difference (Fig. 2b, Table S6A).

The pairwise comparison of band 2 intensity revealed that it is highly significantly increased in P + NS, P + S, and P + RS groups when compared to healthy group. Band 2 showed significant difference in intensity between P + NS and P + S, while P + NS and P + RS showed highly significant difference. Smoker and reverse-smoker groups did not show any significant difference (Fig. 2c, Table S6B).

The pairwise comparison of band 3 intensity revealed no significant difference between P + NS and healthy group. Significant increase was observed between P + S and healthy groups. Highly significant differnce was observed between P + RS and healthy group. Significant difference was observed between P + NS and P + S; P + NS and P + RS groups. Comparision of band 3 intensity between smoker and reverse-smoker groups did not show any significant difference (Fig. 2d, Table S6C).

The quantitative and qualitative analysis of PAs showed that the differences observed in intensities of band 2 and band 3 were found to be more significant when compared to the differences in band 1 intensities and total PA levels between healthy and different periodontitis groups.

### Mass spectrometric and nuclear magnetic resonance (NMR) analysis of separated PA bands

The individual PA bands were identified using high-resolution mass spectrometry (HRMS). Derivatization of PAs with 234 g/mol dansyl chloride resulted in a significant increase in PAs mass. The presence of number of hydrogens and carbon was determined using 1H NMR and 13C NMR spectrometry.

### Identification of band 1

**Fig. 3:**
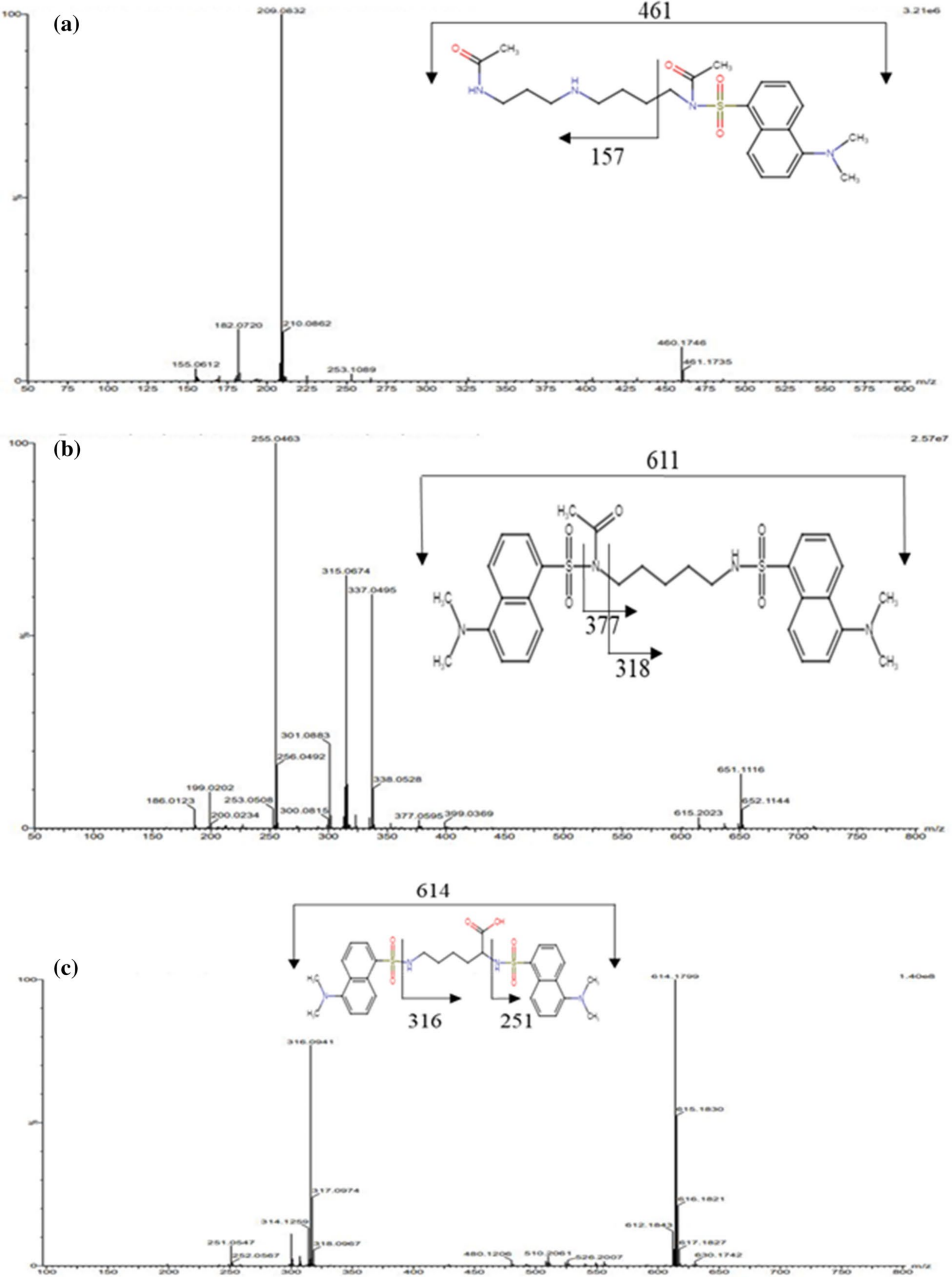
Fragmentation scheme and mass spectrometry (MS) spectrum of dansylated polyamines of Band 1 **a** Band 2 **b**, and Band 3 **c**

The HRMS of dansylated band 1, yielded a peak at m/z 461[M-2] corresponding to the mass of 463 due N1, N8-diacetyl spermidine. The peak m/z at 155 corresponds to the fragment generated due to cleavage between methylene carbon adjacent to N8 acetylated Position [M-dansyl sulphur-CH_2_-NH-CH_3_CO], the peak m/z at 210 could be due to cleavage between N and sulphur of dansyl group with loss of oxygen from either of the acetyl group [M-2H-Dansyl sulfur-O] (Fig. 3a). The band's 1H NMR showed peaks at 1.3 ppm and 2–3, which were attributed to methylene protons between two nitrogens (NH-CH_2_-CH_2_-CH_2_-NH) and methylene protons adjacent to nitrogen (Fig. S1). Furthermore, the peak at 7–8 ppm corresponded to the aromatic hydrogens of dansyl chloride, while the peak at 4.0 ppm corresponded to protons attached to nitrogen. The band's 1H NMR showed peaks at 1.3 ppm and 2–3, which were attributed to methylene protons between two nitrogens (NH-CH_2_-CH_2_-CH_2_-NH) and methylene protons adjacent to nitrogen (Fig. S1). In addition, the peak at 7–8 ppm corresponded to the aromatic hydrogens of dansyl chloride, while the peak at 4.0 ppm corresponded to the protons attached to the nitrogen. The analysis of band 1 by 13C NMR showed peaks at 21.09, 24.87, 29.58, 30.96, 33.17, 36.03, and 39.45 ppm assigned for CH_2._ Peak observed at 155.68, 157.60, and 160.47 ppm were due to the aromatic carbon of dansyl chloride. Peak at 181.70 and 185.86 ppm showed the presence of two carbonyl group (C=O) (Fig. S2). The mass spectrum, 1 H NMR and 13C NMR of band 1 validated the band as N1, N8-diacetyl spermidine. Band 1 showed significant increase in N1, N8-diacetyl spermidine in P + RS and highly significant increase in P + NS.

### Identification of band 2

Dansyl PA band 2 produced a single protonated molecular ion peak at m/z 611, which corresponded to *N*-acetyl cadaverine with two dansyl groups (M + H + 2 dansyl) (Fig. 3b). The fragment generated by the cleavage of the bond between the methyl group of dansyl cadaverine and the *N*-acetyl group corresponds to the peak m/z 315. The mass of *N*-acetyl cadaverine (M + H + dansyl + acetaldehyde) corresponded to the peak at m/z 377. The loss of one dansyl and acetyl group from *N*-acetyl cadaverine resulted in the peak at 337.2 [M + 2]. The presence of hydrogens and carbons in dansylated PA bands 2 was investigated and confirmed using 1H and 13C NMR (Fig. S3, Fig. S4). These findings show that band 2 is *N*-acetyl cadaverine.

### Identification of band 3

Dansyl PA band 3 mass spectra revealed a molecular ion base peak at m/z 614 corresponding to the lysine mass with two dansyl groups (M + H + dansyl) (Fig. 3c). In addition to this, we observed significant peaks at m/z 251 and 317. The first cleavage at m/z 251 could be between dansyl sulfur and amine (M + H-dansyl-SO_2_), and the second cleavage at m/z 317 was due to cleavage at alpha carbon and dansyl amine with carboxylic group loss (M + H-dansyl-COOH). The presence and nature of hydrogens and carbons in dansylated PA bands 3 were investigated and confirmed using 1H NMR and 13 C NMR (Fig. S5, Fig.S6). Band 3 was identified as lysine.

### Antioxidant potential

**Fig. 4:**
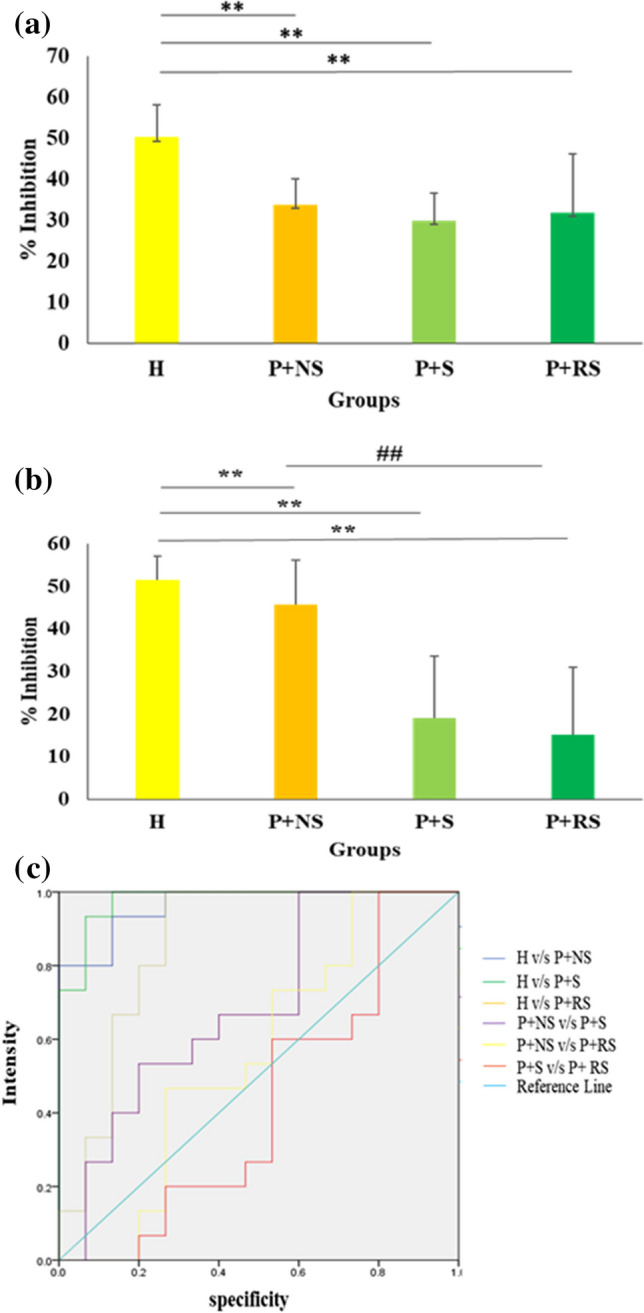
Determination of antioxidant activity in saliva sample **a** DPPH, **b** SOD, **c** Receiver operating characteristic (ROC) curve for screening ability of DPPH; X-axis indicates the study group, Y-axis indicate the measurement antioxidant activity in Unit/µl. * Indicates significant difference (*p* < 0.05). ** Indicates highly significant difference (*p* < 0.01). ## indicates *p* < 0.01 between non-smoker and smoker groups

Significant increase in acetylated PAs; N1, N8-diacetylspermdine and *N*-acetyl cadaverine were shown earlier to be involved in increasing the oxidative stress in different cell types. Therefore, the level of oxidative stress was checked by DPPH and SOD assays in saliva of H, P + NS, P + S, P + RS groups. DPPH results showed the mean levels of % inhibition in the saliva of P + NS, P + S, and P + RS were decreased significantly compared to the healthy group. Intergroup comparison of % inhibition observed a statistically significant decrease in P + NS, P + S, and P + RS respectively compared to healthy group (*p* < 0.05) (Fig. 4a, Table. S7, Table S8). Similarly, the mean level of SOD activity in the saliva of P + NS, P + S, and P + RS were decreased significantly compared to the healthy group. Intergroup comparison showed a statistically significant decrease in P + NS, P + S, and P + RS respectively compared to healthy (*p* < 0.05) (Fig. 4b, Table. S9).

Receiver operating characteristic curve was used to calculate the screening ability of DPPH for periodontitis. It was observed with reference to healthy individuals, polyamines had a sensitivity of 93.3% and specificity of 13.3% in non-smokers with periodontitis, while in smokers with periodontitis the sensitivity was 93.3% and specificity was 67% and in reverse smokers with periodontitis the sensitivity was 46.7% and specificity was 53.3% for the detection of periodontitis (Fig. .4c, Table S10).

## Discussion

Early diagnosis of periodontal disease is of paramount importance, especially in smokers. Active periodontal disease diagnosis and distinguishing patients at risk of periodontal disease is still a challenge. Salivary diagnostic tests can be employed as a chair-side diagnostic tool for early detection of disease(Giannobile et al. 2009). Smoking is an independent risk factor for periodontal disease. It affects the subgingival microflora, the human immune response, and increases the oxidative stress that leads to increased inflammation and destruction of the supporting tissues of the tooth (Michael et al. 2007)*.* PAs are involved in inflammation, bone formation, and cell proliferation, all of which are important in periodontitis patients. The variation of PAs in periodontitis patients especially in smokers was not properly evaluated to date. Hence, this study aimed to analyse the diagnostic ability of salivary PAs levels in reverse smokers and smokers of periodontitis.

In the current study, an intergroup comparison of PAs revealed a statistically significant difference (*p* < 0.018), with reverse smokers periodontitis group having the highest levels of PAs, followed by smokers with periodontitis, non-smokers with periodontitis, and healthy individuals. PA levels in edematous and granulomatous tissue were elevated in chronic (adjuvant-induced arthritis) and sub-chronic inflammatory conditions in mice models (Chakradhar and Naik 2007). The increase in PAs observed during inflammation is most likely due to the activation of ornithine decarboxylase, a rate-limiting enzyme in PAs (Zhang et al. 2000). Depending on the definition of disease and smoking exposure, the risk of periodontal destruction for a smoker is 5 to 20-fold higher than for a non-smoker. In line with this, PAs were found to be significantly higher in reverse smokers with periodontitis in the current study, indicating higher levels of inflammation and oxidative stress.

Qualitative analysis of PAs showed three prominent bands which showed differences between the groups. Band 1, was identified as N1,N8-diacetylspermidine. It showed significant differences between healthy and non-smoker periodontitis group. It is involved in cell proliferation. The increased levels of it could be for the maintenance of cellular homeostasis of PA levels. Acetylated PAs contribute more to the oxidative stress through spermidine/spermine-N1-acetyltransferase and polyamine oxidase enzymes (Pegg et al. 2008). However, significant differences were not observed between healthy and smoker groups.

Band 2 was identified as N acetylcadaverine. Highly significant increase in its levels was observed in different periodontitis groups when compared to healthy group. It was found to be elevtated more in non-smoker groups when compared to smoker gorups. The increase in N acetylcadaverine is tightly associated as putative component of oral malodor and butyrate producing microbiome which leads to enhanced GCF substrate. This would slowly destroy the periodontium (Goldberg et al. 1994). Similar result was obtained in earlier studies associated with periodontitis which results in tooth loss (Andorfer et al. 2021; Kuboniwa et al. 2016).

Band 3 was identifed as lysine. Highly significant increase in its levels was observed in different periodontitis groups when compared to healthy group. Significant differences were found between non-smoker and smoker periodontitis groups. Elevated levels of lysine in the present study correlated with the study that showed. Lysine were significantly higher in the group of deep-pocket sites in comparison to the group of healthy sites and moderate-pocket sites (Ozeki et al. 2016). involvment of lysine in gingivitis and periodontitis (Levine and Lohinai 2021). The failure of GCF exudation in smokers results in the junctional epithelial attachment becoming deprived of amino acids including lysine.

The N1, N8-diacetyl spermidine, and *N*-acetyl cadaverine levels showed highly significant increase in periodontitis. The statistically significant decreased levels of DPPH and SOD in smokers with periodontitis when compared to other groups could have helped in tissue degradation as reported earlier with tobacco stress (Nosratabadi et al. 2012). The relationship between PAs and oxidative stress was reported to be bidirectional. PA synthesis is in turn induced by oxidative stress, as increase in expression of PA concentration-dependent genes are involved in protecting against ROS stress and repairing damage (Vrijsen et al. 2023). Nurcan Göktürk in 2022 has also reported increased oxidative stress caused an increase in PAs levels. The antioxidant potential between healthy and periodontitis groups was significant posing it as a better biomarker. However, DPPH assay did not show significant difference among non-smoker, smoker and reverse smoker periodontitis (Table S8). Therefore, we suspect that this cannot be used for checking the severity of the disease due to smoking. But, the levels of *N* acetyl cadaverine and lysine showed significant differences not only between healthy and periodontitis groups but also among different periodontitis groups. Hence, a combined assessment of *N* acetyl cadaverine and lysine will be a better biomarker for smoker periodontitis. The present study suggests that quantitative analysis of *N*-acetyl cadaverine and lysine could serve as potential biomarkers for individuals with periodontitis who smoke, including those who engage in reverse smoking.

## Conclusion

In conclusion, PAs levels are elevated in saliva samples of smoker periodontitis groups indicating their active involvement in severity of the disease. The present study shows a relationship between different periodontitis groups and PA levels. Therefore, the study shows that instead of single polyamine as a biomarker, combined analyses of lysine, and *N*-acetyl cadaverine can be used as an easy tool for determination of levels of tissue degradation and diagnosis of smoker periodontitis patients.

**Fig. 5:**
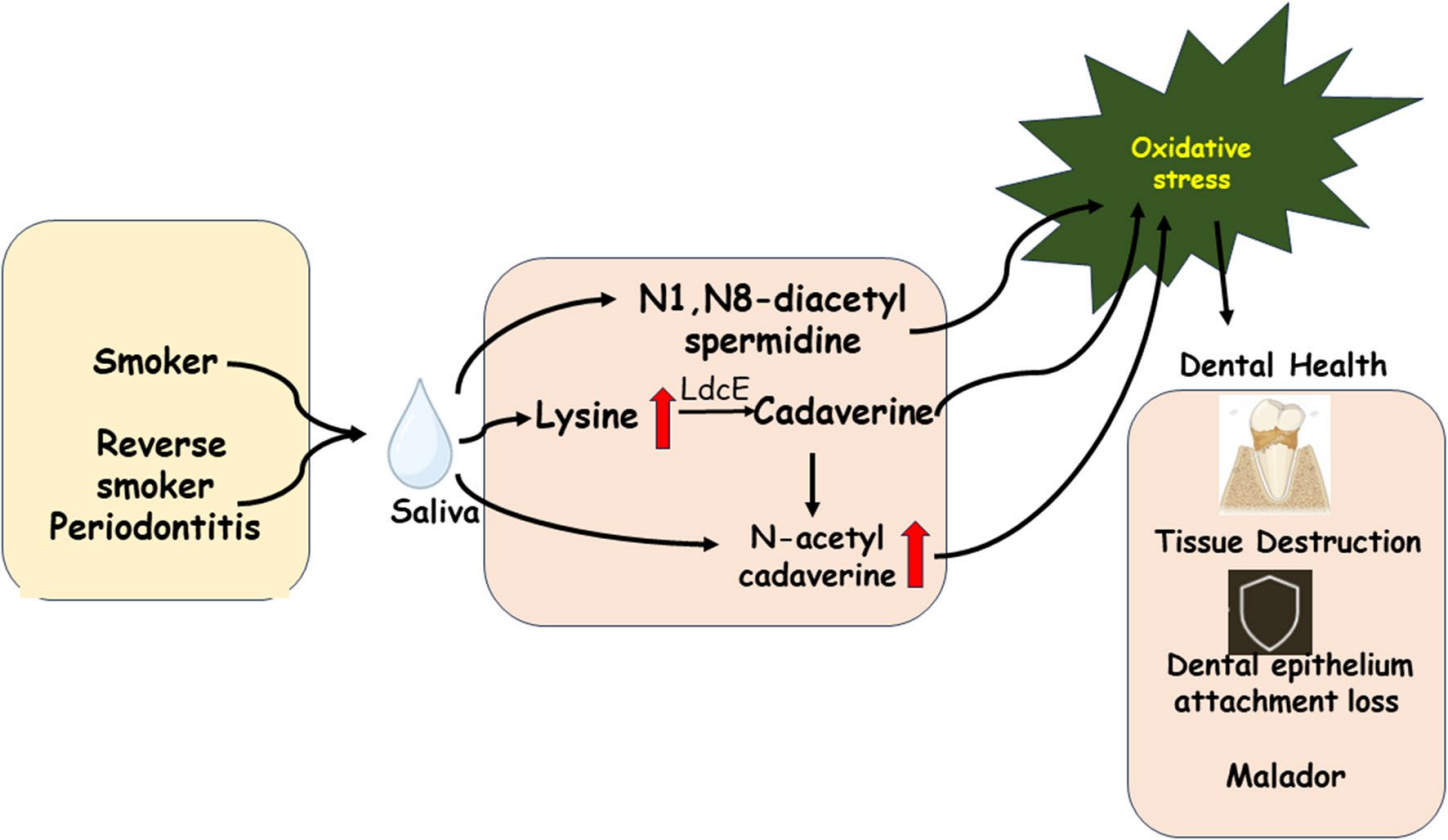
Schematic representation showing the roles of polyamines and their derivatives in the smoker periodontitis that promotes tissue degradation, oral malodor and oxidative stress. Red arrows indicate elevation in the levels of the polyamines

The Fig. 5 represents overall conclusion of the manuscript.

## Supplementary Information

Below is the link to the electronic supplementary material.

Figure S1 1H NMR (nuclear magnetic resonance) analysis of dansylated PAs of Band1 pooled from TLC

Figure S2 13C NMR (nuclear magnetic resonance) analysis of dansylated PAs of Band 1 pooled from TLC

Figure S3 1H NMR (nuclear magnetic resonance) analysis of dansylated PAs of Band 2 pooled from TLC

Figure S4 13C NMR (nuclear magnetic resonance) analysis of dansylated PAs of Band 2 pooled from TLC

Figure S5 1H NMR (nuclear magnetic resonance) analysis of dansylated PAs of Band 3 pooled from TLC

Figure S6 13C NMR (nuclear magnetic resonance) analysis of dansylated PAs of Band 3 pooled from TLC

Figure S7 Mass spectrometry (MS) spectrum of dansylated polyamines of Band 4

Figure S8 1H NMR (nuclear magnetic resonance) analysis of dansylated PAs of Band 4 pooled from TLC

Figure S9 13C NMR (nuclear magnetic resonance) analysis of dansylated PAs of Band 4 pooled from TLC

Supplementary file10 (DOCX 14 KB)

Supplementary file11 (DOCX 15 KB)

Supplementary file12 (DOCX 15 KB)

Supplementary file13 (DOCX 15 KB)

Supplementary file14 (DOCX 17 KB)

Supplementary file15 (DOCX 17 KB)

Supplementary file16 (DOCX 14 KB)

Supplementary file17 (DOCX 15 KB)

Supplementary file18 (DOCX 14 KB)

Supplementary file19 (DOCX 16 KB)

## Data Availability

No datasets were generated or analysed during the current study.

## Abbreviations

PA: Polyamine
PCA: Perchloric acid
S + P: Smoker and periodontitis
P + NS: Non-smoker and periodontitis
P + RS: Reverse smoker and periodontitis
DNA: Deoxyribonucleic acid
ROC: Receiver operating characteristic

## References

[CR1] Andörfer L, Holtfreter B, Weiss S, Matthes R, Pitchika V, Schmidt CO, Samietz S, Kastenmüller G, Nauck M, Völker U, Völzke H, Csonka LN, Suhre K, Pietzner M, Kocher T (2021) Salivary metabolites associated with a 5-year tooth loss identified in a population-based setting. BMC Med 19(1):161. 10.1186/s12916-021-02035-z 34256740 10.1186/s12916-021-02035-z PMC8278731

[CR2] Bachtiar EW, Gusliana DS, Bachtiar BM (2021) Correlation between the extent of smoking, salivary protein profiles, and dental caries in young adult smokers. Saudi Dental J 33(7):533–537. 10.1016/j.sdentj.2020.09.002 10.1016/j.sdentj.2020.09.002 PMC8589578 34803297

[CR3] Beauchamp C, Fridovich I (1971) Superoxide dismutase: improved assays and an assay applicable to acrylamide gels. Anal Biochem 44(1):276–287. 10.1016/0003-2697(71)90370-8 4943714 10.1016/0003-2697(71)90370-8

[CR4] Brand-Williams W, Cuvelier ME, Berset C (1995) Use of a free radical method to evaluate antioxidant activity. LWT Food Sci Technol 28(1):25–30. 10.1016/S0023-6438(95)80008-5

[CR5] Chakradhar VL, Naik SR (2007) Polyamines in inflammation and their modulation by conventional anti-inflammatory drugs. Indian J Exp Biol 45(7):649–653 17821863

[CR6] Cortelli SC, Máximo PDM, Peralta FS, Silva RAD, Rovai ES, Costa FO, Aquino DR, Rodrigues E, Cortelli JR (2021) Salivary nitrite and systemic biomarkers in obese individuals with periodontitis submitted to FMD. Brazilian Dental Journal 32:27–36 34614058 10.1590/0103-6440202103782

[CR7] D’Aiuto F, Sabbah W, Netuveli G, Donos N, Hingorani AD, Deanfield J, Tsakos G (2008) Association of the metabolic syndrome with severe periodontitis in a large us population-based survey. J Clin Endocrinol Metab 93(10):3989–3994 18682518 10.1210/jc.2007-2522

[CR8] De Smit MJ, Westra J, Brouwer E, Janssen KMJ, Vissink A, Van Winkelhoff AJ (2015) Commentary: periodontitis and rheumatoid arthritis: what do we know? J Periodontol 86(9):1013–1019. 10.1902/jop.2015.150088 25968957 10.1902/jop.2015.150088

[CR9] Giannobile WV, Beikler T, Kinney JS, Ramseier CA, Morelli T, Wong DT (2009) Saliva as a diagnostic tool for periodontal disease: current state and future directions. Periodontol 2000(50):52 10.1111/j.1600-0757.2008.00288.x PMC5695225 19388953

[CR10] Göktürk N, Şahin H, Bayramoğlu F, Çakıcı Ç, Büyükuslu N, Yiğit P, Yiğitbaşı T (2022) Could the increase in oxidative stress be the reason for the increased polyamine levels in diabetic obese and non-diabetic obese patients? ACTA Pharmaceutica Sciencia. 60(3):301

[CR11] Goldberg S, Kozlovsky A, Gordon D, Gelernter I, Sintov A, Rosenberg M (1994) Cadaverine as a putative component of oral malodor. J Dent Res 73(6):1168–1172. 10.1177/00220345940730060701 8046106 10.1177/00220345940730060701

[CR12] Haber J, Wattles J, Crowley M, Mandell R, Joshipura K, Kent RL (1993) Evidence for cigarette smoking as a major risk factor for periodontitis. J Periodontol 64(1):16–23. 10.1902/jop.1993.64.1.16 8426285 10.1902/jop.1993.64.1.16

[CR13] Henderson Pozzi M, Gawandi V, Fitzpatrick PF (2009) pH Dependence of a mammalian polyamine oxidase: insights into substrate specificity and the role of lysine 315. Biochemistry 48(7):1508–1516. 10.1021/bi802227m 19199575 10.1021/bi802227m PMC2752350

[CR14] Igarashi K, Kashiwagi K (2018) Effects of polyamines on protein synthesis and growth of escherichia coli. J Biol Chem 293(48):18702–18709. 10.1074/jbc.TM118.003465 30108177 10.1074/jbc.TM118.003465 PMC6290148

[CR15] Ishida H, Iwayama Y, Daikuhara Y (1983) Changes in polyamine metabolism during experimental periodontitis in dogs and the role of putrescine in recovery. Arch Oral Biol 28(1):51–60. 10.1016/0003-9969(83)90026-2 6575737 10.1016/0003-9969(83)90026-2

[CR16] Kolte A, Kolte R, Laddha R (2012) Effect of smoking on salivary composition and periodontal status. J Indian Soc Periodontol 16(3):350. 10.4103/0972-124X.100909 23162327 10.4103/0972-124X.100909 PMC3498702

[CR17] Kuboniwa M, Sakanaka A, Hashino E, Bamba T, Fukusaki E, Amano A (2016) Prediction of periodontal inflammation via metabolic profiling of saliva. J Dent Res 95(12):1381–1386. 10.1177/0022034516661142 27470067 10.1177/0022034516661142

[CR18] Levine M, Lohinai ZM (2021) Resolving the contradictory functions of lysine decarboxylase and butyrate in periodontal and intestinal diseases. J Clin Med 10(11):2360. 10.3390/jcm10112360 34072136 10.3390/jcm10112360 PMC8198195

[CR19] Lim HK, Rahim AB, Leo VI, Das S, Lim TC, Uemura T, Igarashi K, Common J, Vardy LA (2018) Polyamine regulator AMD1 promotes cell migration in epidermal wound healing. J Investig Dermatol 138(12):2653–2665. 10.1016/j.jid.2018.05.029 29906410 10.1016/j.jid.2018.05.029

[CR20] Lima A, Didugu BGL, Chunduri AR, Rajan R, Jha A, Mamillapalli A (2023) Thermal tolerance role of novel polyamine, caldopentamine, identified in fifth instar *Bombyx mori*. Amino Acids 55(2):287–298. 10.1007/s00726-022-03226-5 36562834 10.1007/s00726-022-03226-5

[CR21] Michael GN, Henry HT, Perry RK, Fermin AC (2018) Caranza's clinical periodontology. Saunders Elsevier, St. Louis, Missouri

[CR22] Mustafavi SH, Naghdi Badi H, Sękara A, Mehrafarin A, Janda T, Ghorbanpour M, Rafiee H (2018) Polyamines and their possible mechanisms involved in plant physiological processes and elicitation of secondary metabolites. Acta Physiol Plant 40(6):102. 10.1007/s11738-018-2671-2

[CR23] Nosratabadi SF, Sariri R, Yaghmaei P, Taheri M, Ghadimi A, Ghafoori H (2012) Alternations of antioxidant activity in saliva in smokers. J Phys Theor Chem 8:305–310

[CR24] Ozeki M, Nozaki T, Aoki J, Bamba T, Jensen KR, Murakami S, Toyoda M (2016) Metabolomic analysis of gingival crevicular fluid using gas chromatography/mass spectrometry. Mass Spectrom (Tokyo, Japan) 5(1):A0047. 10.5702/massspectrometry.A0047 10.5702/massspectrometry.A0047 PMC4926765 27446770

[CR25] Papapanou PN, Sanz M, Buduneli N, Dietrich T, Feres M, Fine DH, Flemmig TF, Garcia R, Giannobile WV, Graziani F (2018) Periodontitis: consensus report of workgroup 2 of the 2017 World workshop on the classification of periodontal and peri-implant diseases and conditions. J Periodontol 89:S173–S182 29926951 10.1002/JPER.17-0721

[CR26] Pegg AE (2008) Spermidine/spermine-N 1-acetyltransferase: a key metabolic regulator. Am J Physiol-Endocrinol Metabolism 294(6):E995–E1010 10.1152/ajpendo.90217.2008 18349109

[CR27] Pindborg JJ, Mehta FS, Gupta PC, Daftary DK, Smith CJ (1971) Reverse smoking in Andhra Pradesh, India: a study of palatal lesions among 10,169 villagers. Br J Cancer 25(1):10–20 5581290 10.1038/bjc.1971.2 PMC2008558

[CR28] Sakanaka A, Kuboniwa M, Hashino E, Bamba T, Fukusaki E, Amano A (2017) Distinct signatures of dental plaque metabolic byproducts dictated by periodontal inflammatory status. Sci Rep 7(1):42818. 10.1038/srep42818 28220901 10.1038/srep42818 PMC5318866

[CR29] Sanz M, Marco Del Castillo A, Jepsen S, Gonzalez-Juanatey JR, D’Aiuto F, Bouchard P, Chapple I, Dietrich T, Gotsman I, Graziani F, Herrera D, Loos B, Madianos P, Michel J, Perel P, Pieske B, Shapira L, Shechter M, Tonetti M, Wimmer G (2020) Periodontitis and cardiovascular diseases: consensus report. J Clinical Periodontol 47(3):268–288. 10.1111/jcpe.13189 32011025 10.1111/jcpe.13189 PMC7027895

[CR30] Vrijsen S, Houdou M, Cascalho A, Eggermont J, Vangheluwe P (2023) Polyamines in parkinson’s disease: balancing between neurotoxicity and neuroprotection. Annu Rev Biochem 92(1):435–464. 10.1146/annurev-biochem-071322-021330 37018845 10.1146/annurev-biochem-071322-021330

[CR31] WHO FACT SHEET (2022) https://www.who.int/news-room/fact-sheets/detail/tobacco Accessed 2 June 2023 (n.d.) dataset

[CR32] Yamamoto T, Hinoi E, Fujita H, Iezaki T, Takahata Y, Takamori M, Yoneda Y (2012) The natural polyamines spermidine and spermine prevent bone loss through preferential disruption of osteoclastic activation in ovariectomized mice: polyamines inhibit osteoclastogenesis. Br J Pharmacol 166(3):1084–1096. 10.1111/j.1476-5381.2012.01856.x 22250848 10.1111/j.1476-5381.2012.01856.x PMC3417431

[CR33] Zhang M, Wang H, Tracey KJ (2000) Regulation of macrophage activation and inflammation by spermine: a new chapter in an old story. Crit Care Med 28(Supplement):N60–N66. 10.1097/00003246-200004001-00007 10807317 10.1097/00003246-200004001-00007

