# Supplementary material for: Combined assessment of lysine and N-acetyl cadaverine levels assist as a potential biomarker of the smoker periodontitis
Source: Amino Acids. 2024 Jun 8;56:41. doi: 10.1007/s00726-024-03396-4 (PMC11162398; doi:10.1007/s00726-024-03396-4)
Supplement: Supplementary file 2 [file 726_2024_3396_MOESM11_ESM.docx]

**Table S2: Pairwise comparison of polyamine between the groups using Tukey’s Test**

| **(A)** | **(B)** | **Mean Difference**  **(A-B)** | **Std. Error** | **p-value** | **95% Confidence Interval** | |
| --- | --- | --- | --- | --- | --- | --- |
|  |  |  |  |  | **Lower Bound** | **Upper Bound** |
| H | P+NS | -.98353 | 2.41708 | 0.977 | -7.3594 | 5.3923 |
| H | P+S | -5.73813 | 2.41708 | 0.093 | -12.1140 | 0.6377 |
| H | P+RS | -6.39498 | 2.41708 | 0.040* | -12.7708 | -0.0191 |
| P+NS | P+S | -4.75460 | 2.41708 | 0.211 | -11.1305 | 1.6213 |
| P+NS | P+RS | -5.41145 | 2.41708 | 0.124 | -11.7873 | 0.9644 |
| P+S | P+RS | -.65685 | 2.41708 | 0.993 | -7.0327 | 5.7190 |

*Statistically significant, Tukey’s Test
