# Supplementary material for: Combined assessment of lysine and N-acetyl cadaverine levels assist as a potential biomarker of the smoker periodontitis
Source: Amino Acids. 2024 Jun 8;56:41. doi: 10.1007/s00726-024-03396-4 (PMC11162398; doi:10.1007/s00726-024-03396-4)
Supplement: Supplementary file 7 [file 726_2024_3396_MOESM16_ESM.docx]

**Table S7: Comparison of DPPH between the groups using one-way ANOVA**

| **Groups** | **Mean** | **Standard deviation** | **F value** | **P value** |
| --- | --- | --- | --- | --- |
| Healthy | 50.1667 | 7.84050 | 14.955 | 0.000* |
| P+NS | 33.8047 | 6.27582 |  |  |
| P+S | 29.8953 | 6.64619 |  |  |
| P+RS | 31.8287 | 14.21102 |  |  |

*Statistically significant
