# Supplementary figures and images for: Combined assessment of lysine and N-acetyl cadaverine levels assist as a potential biomarker of the smoker periodontitis
Source: Amino Acids. 2024 Jun 8;56(1):41. doi: 10.1007/s00726-024-03396-4 (PMC11162398; doi:10.1007/s00726-024-03396-4)

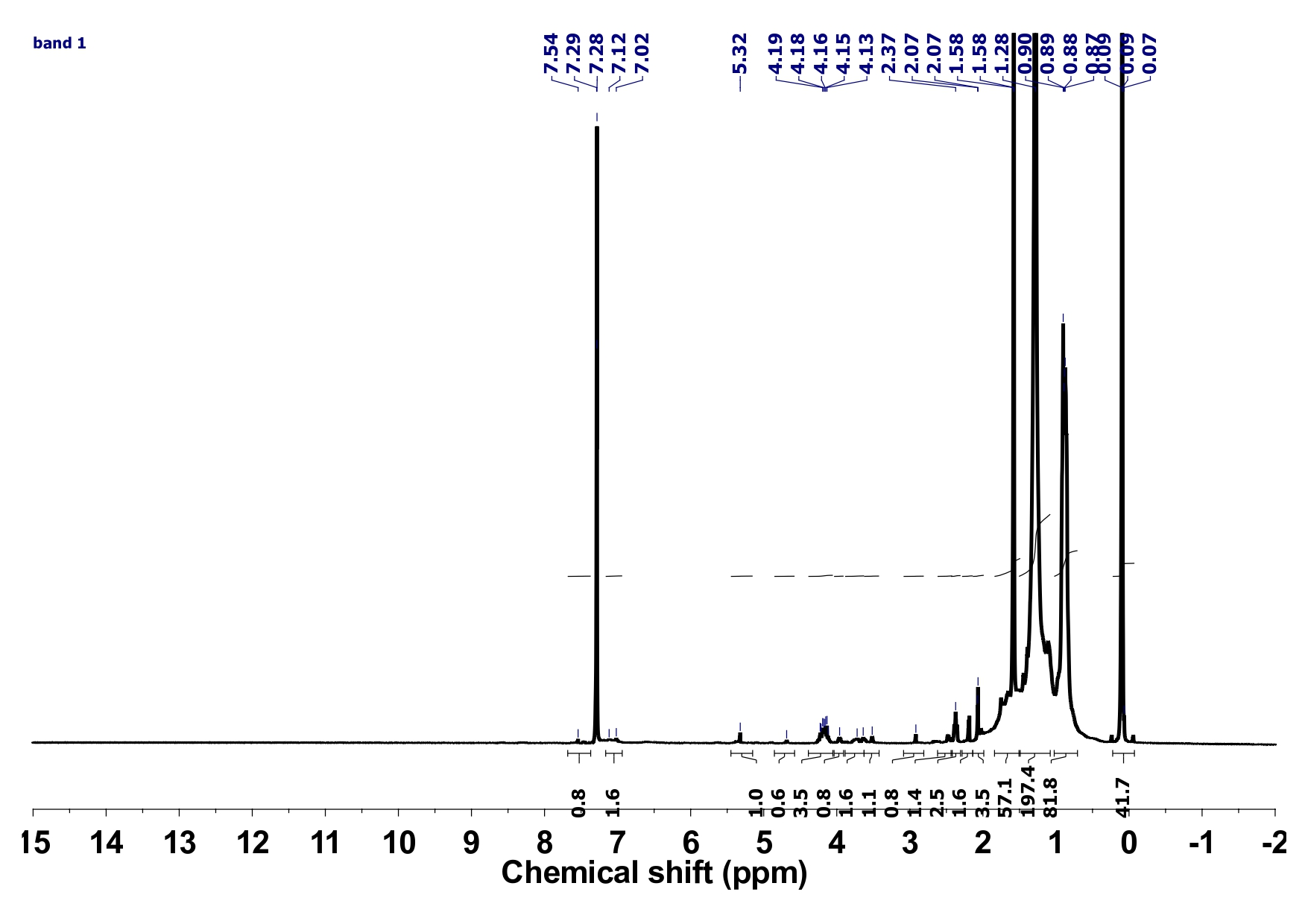

Supplement: Supplementary file 1 — Figure S1 1H NMR (nuclear magnetic resonance) analysis of dansylated PAs of Band1 pooled from TLC [file 726_2024_3396_MOESM1_ESM.tif]

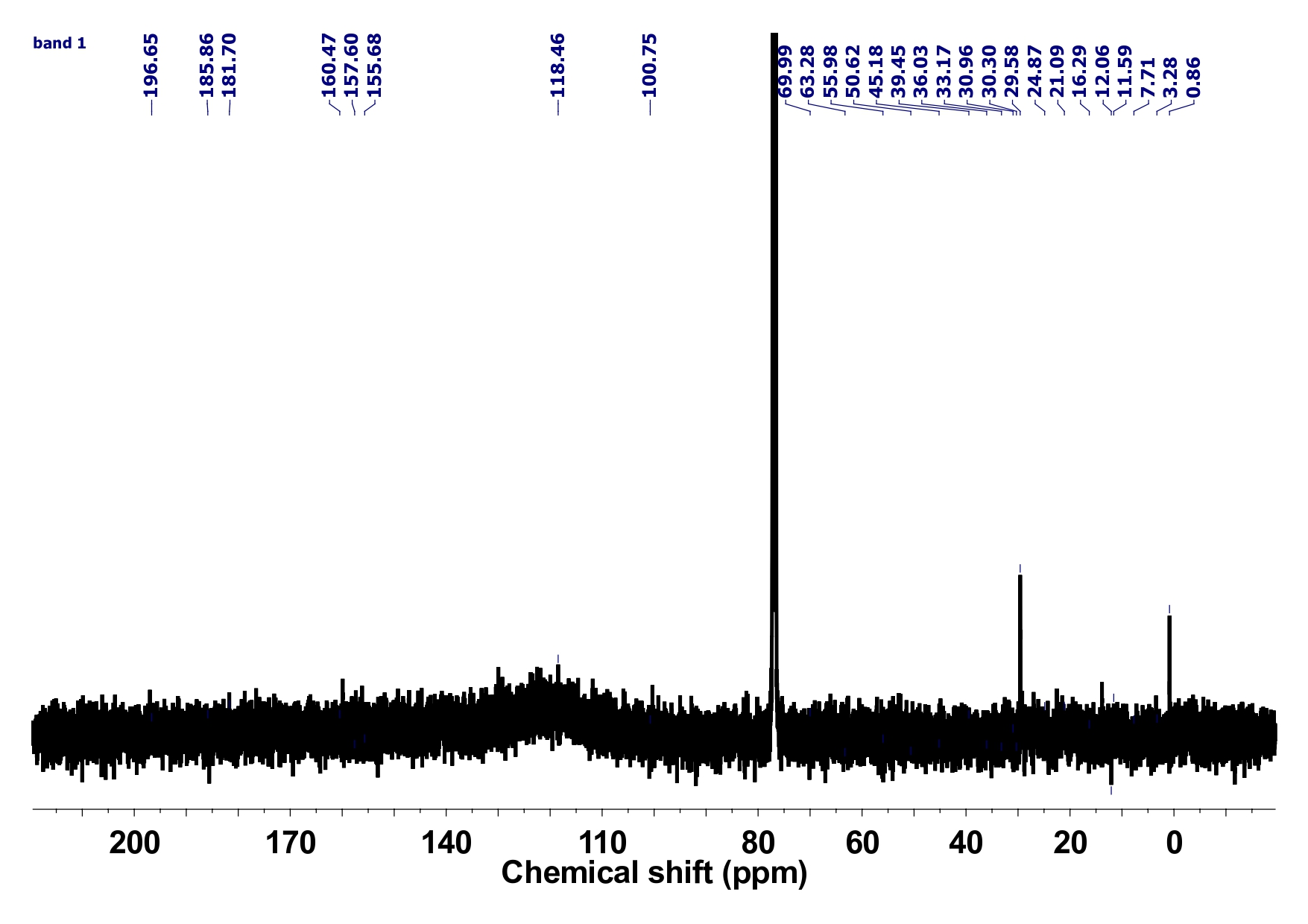

Supplement: Supplementary file 2 — Figure S2 13C NMR (nuclear magnetic resonance) analysis of dansylated PAs of Band 1 pooled from TLC [file 726_2024_3396_MOESM2_ESM.tif]

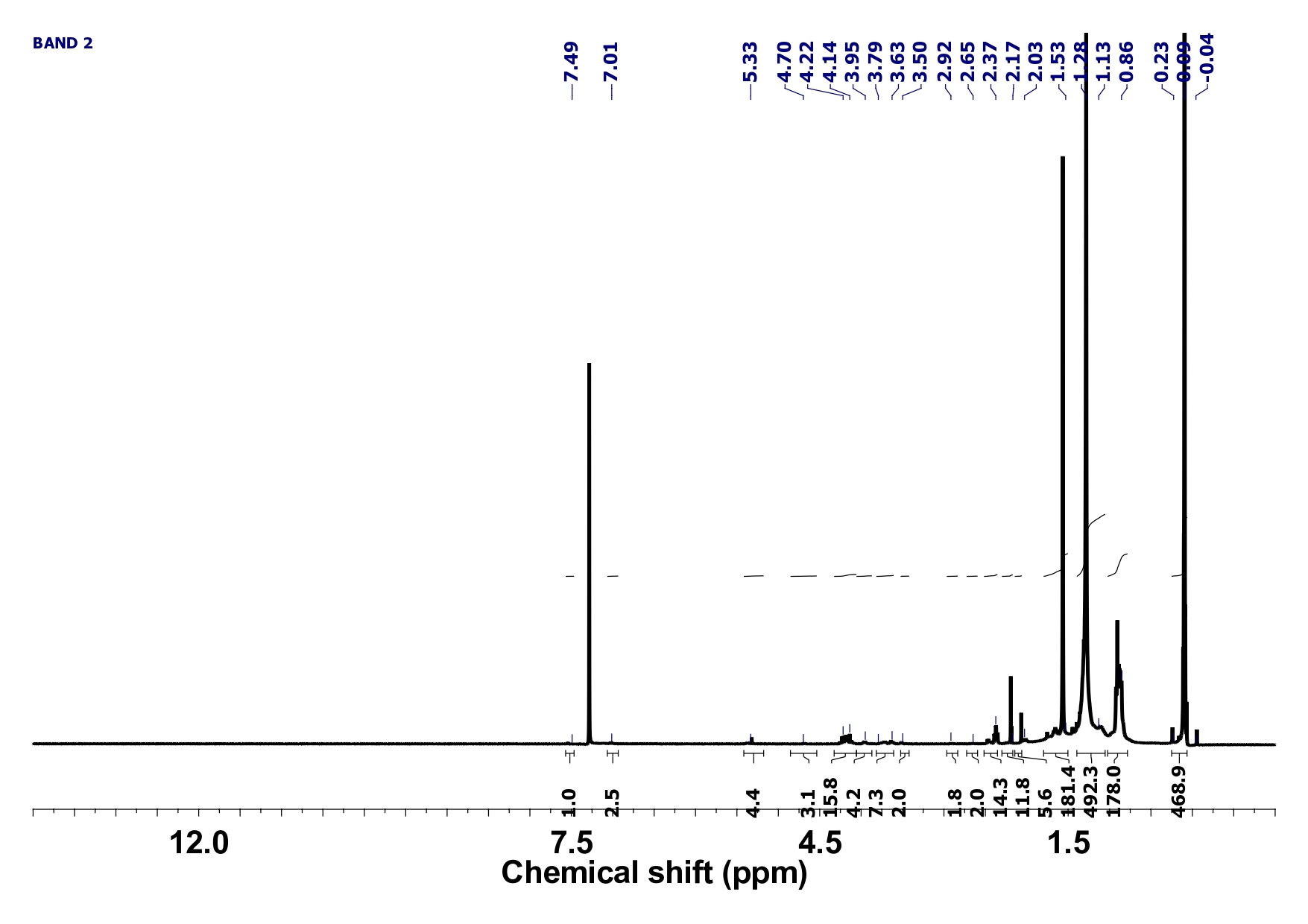

Supplement: Supplementary file 3 — Figure S3 1H NMR (nuclear magnetic resonance) analysis of dansylated PAs of Band 2 pooled from TLC [file 726_2024_3396_MOESM3_ESM.tif]

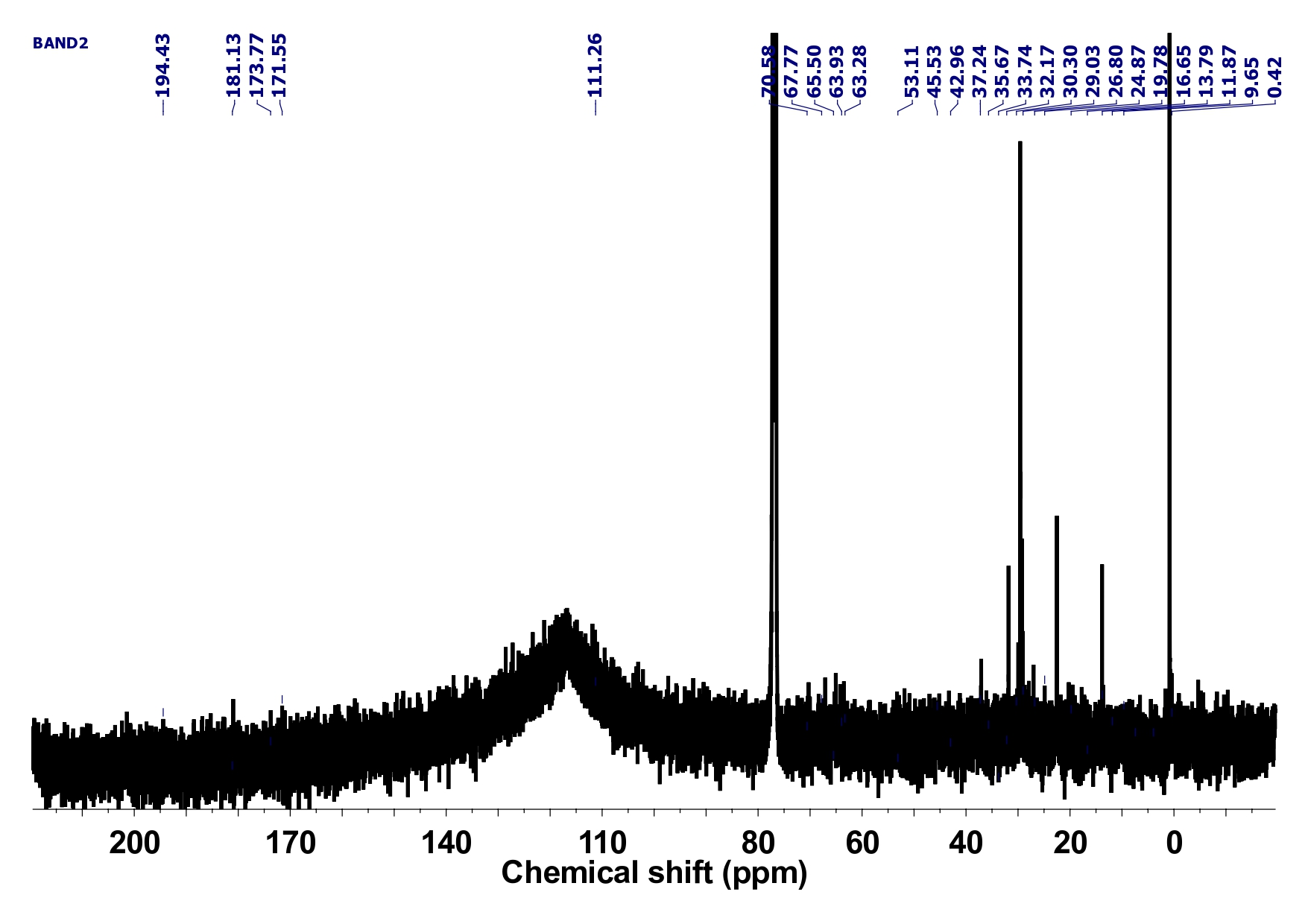

Supplement: Supplementary file 4 — Figure S4 13C NMR (nuclear magnetic resonance) analysis of dansylated PAs of Band 2 pooled from TLC [file 726_2024_3396_MOESM4_ESM.tif]

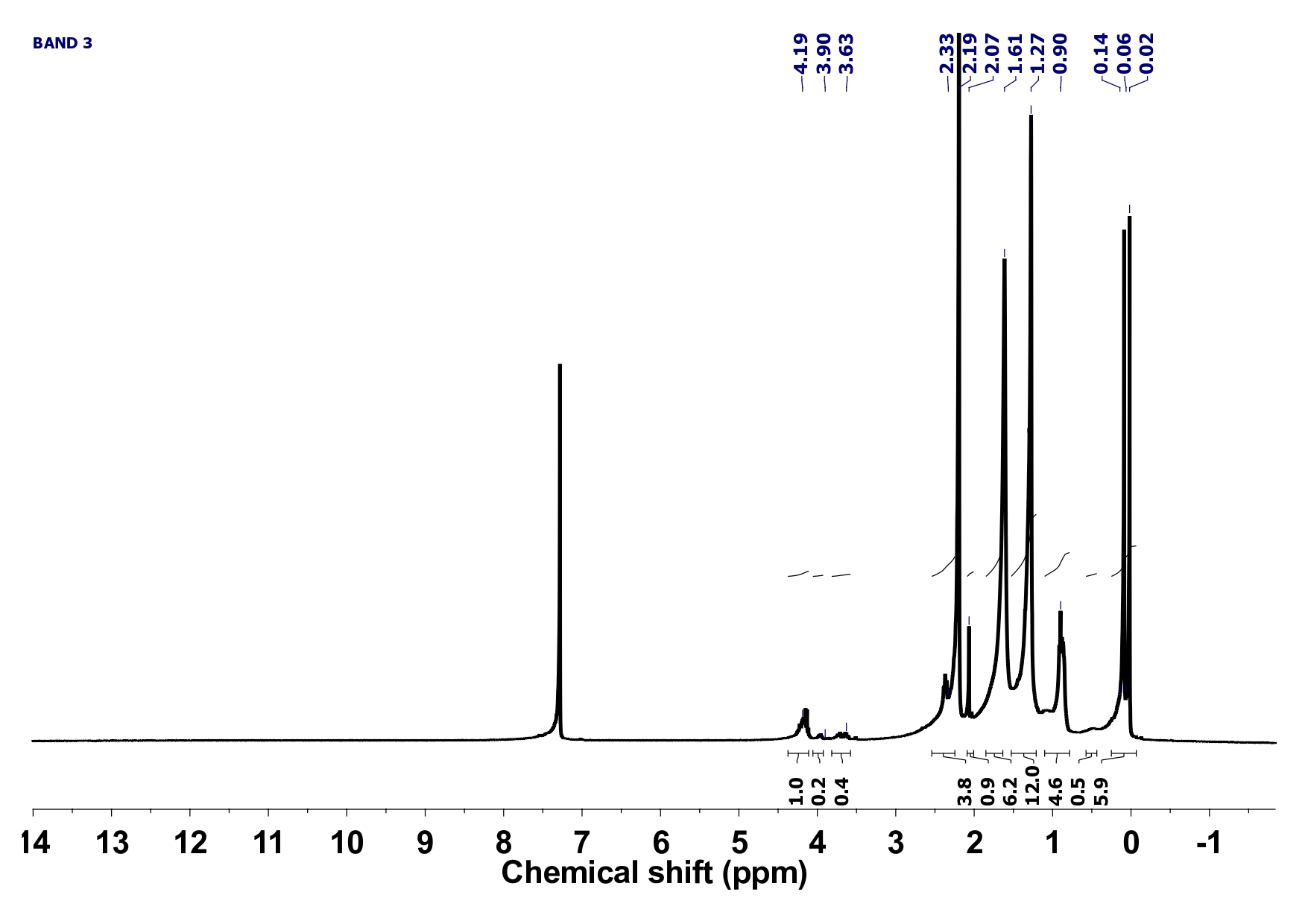

Supplement: Supplementary file 5 — Figure S5 1H NMR (nuclear magnetic resonance) analysis of dansylated PAs of Band 3 pooled from TLC [file 726_2024_3396_MOESM5_ESM.tif]

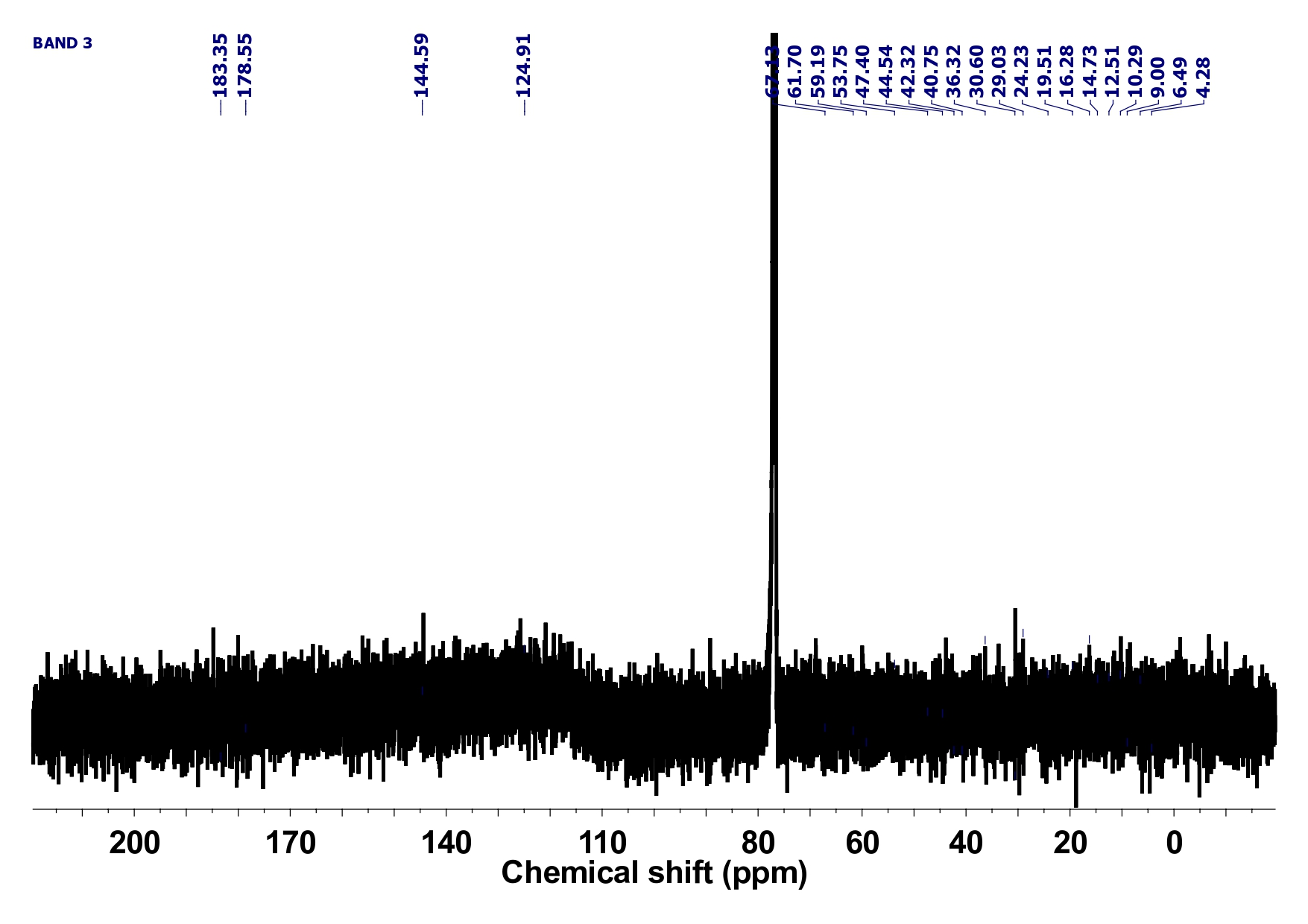

Supplement: Supplementary file 6 — Figure S6 13C NMR (nuclear magnetic resonance) analysis of dansylated PAs of Band 3 pooled from TLC [file 726_2024_3396_MOESM6_ESM.tif]

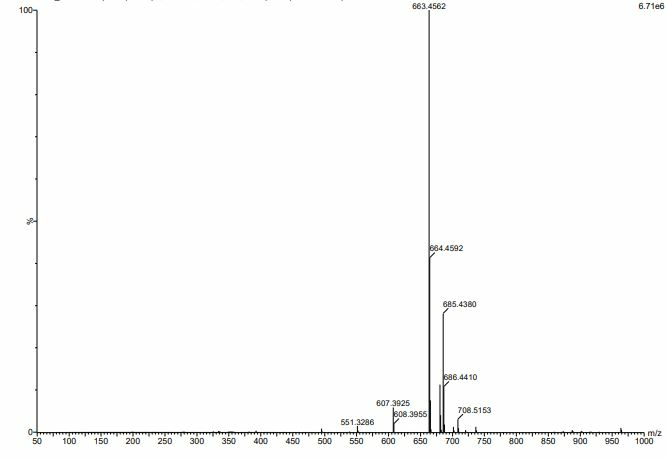

Supplement: Supplementary file 7 — Figure S7 Mass spectrometry (MS) spectrum of dansylated polyamines of Band 4 [file 726_2024_3396_MOESM7_ESM.tif]

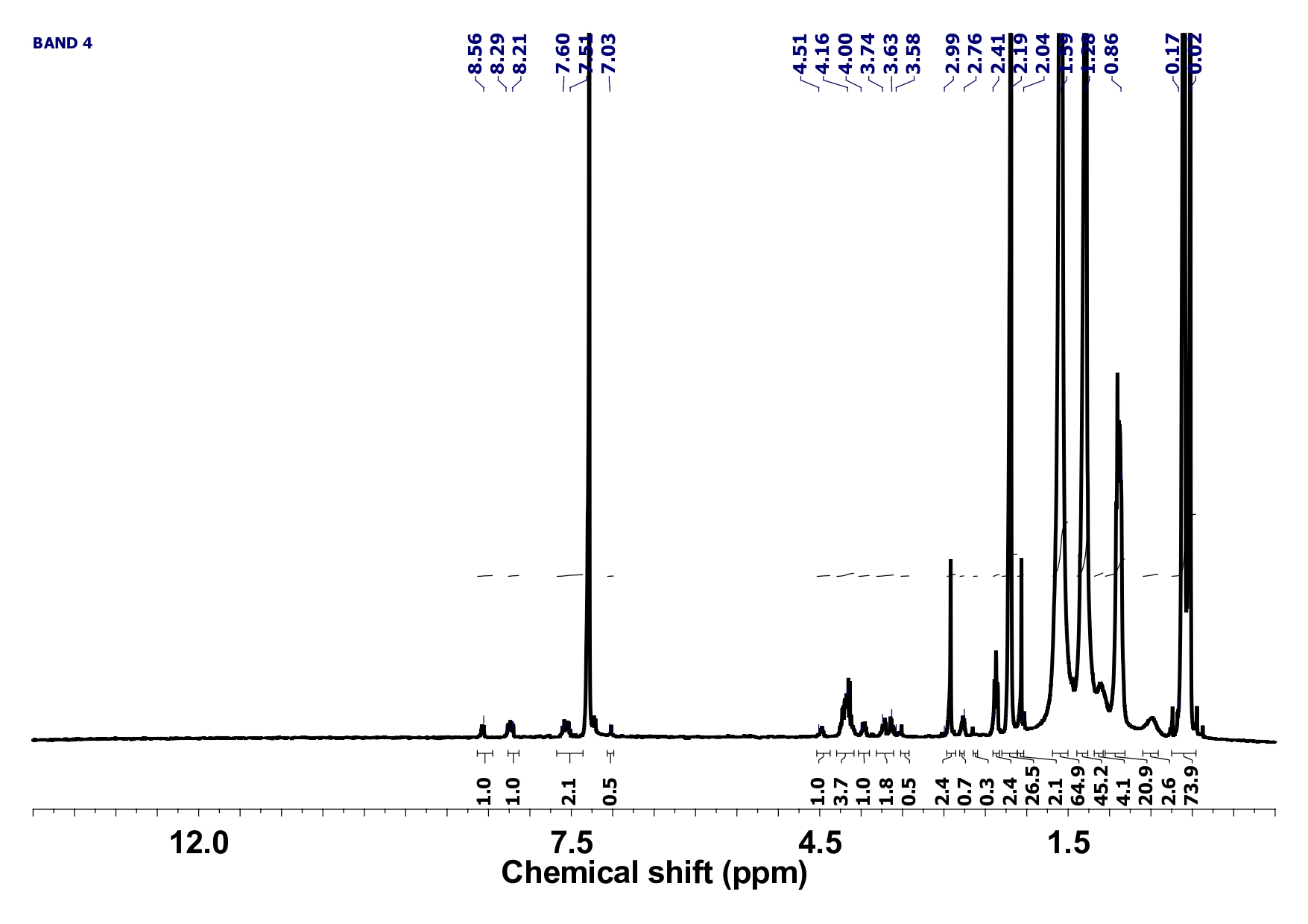

Supplement: Supplementary file 8 — Figure S8 1H NMR (nuclear magnetic resonance) analysis of dansylated PAs of Band 4 pooled from TLC [file 726_2024_3396_MOESM8_ESM.tif]

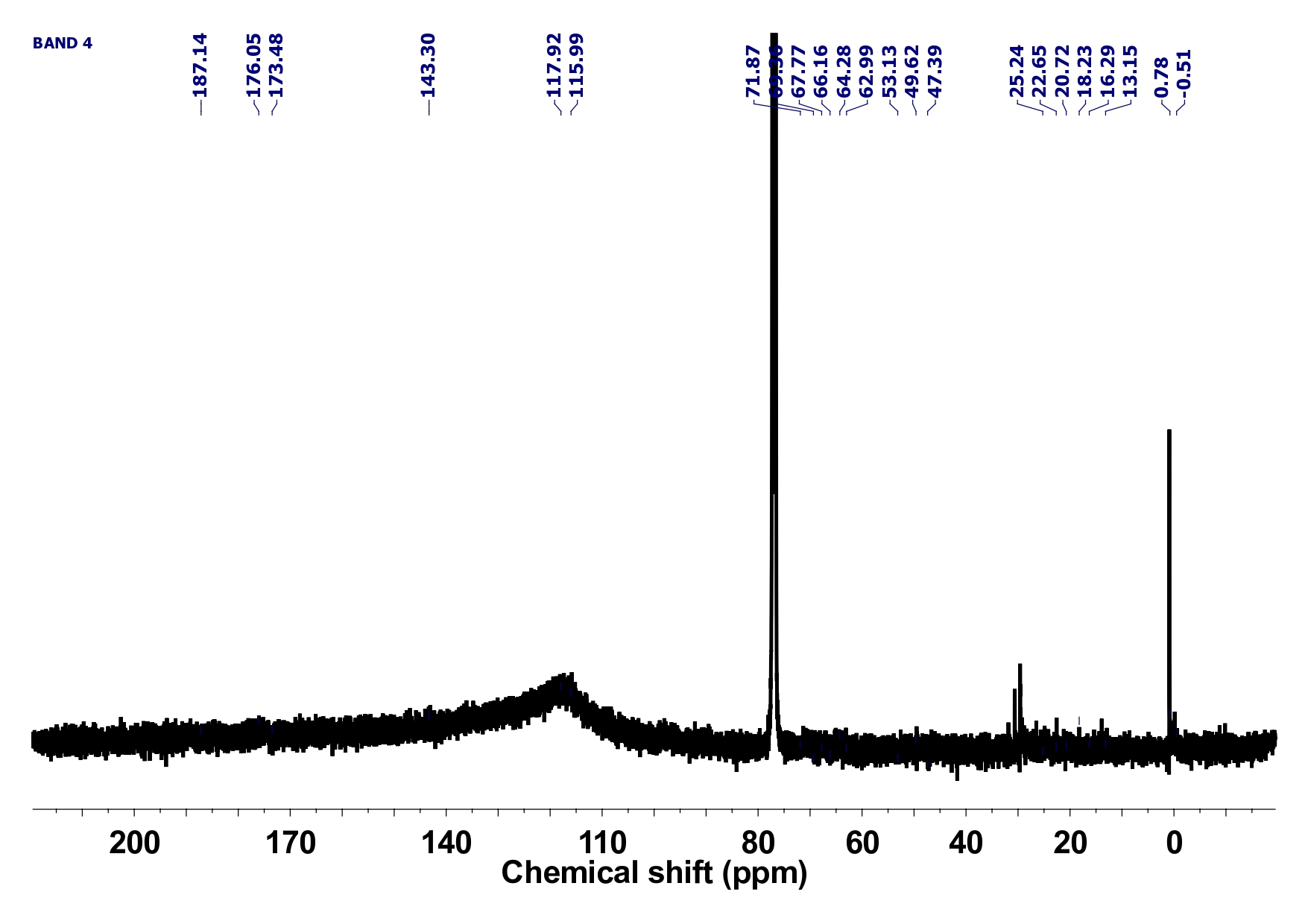

Supplement: Supplementary file 9 — Figure S9 13C NMR (nuclear magnetic resonance) analysis of dansylated PAs of Band 4 pooled from TLC [file 726_2024_3396_MOESM9_ESM.tif]
