# Supplementary material for: Combined assessment of lysine and N-acetyl cadaverine levels assist as a potential biomarker of the smoker periodontitis
Source: Amino Acids. 2024 Jun 8;56(1):41. doi: 10.1007/s00726-024-03396-4 (PMC11162398; doi:10.1007/s00726-024-03396-4)
Supplement: Supplementary file 10 — Supplementary file10 (DOCX 14 KB) [file 726_2024_3396_MOESM10_ESM.docx]

**Table S1: Comparison of Polyamine levels among the groups using one-way ANOVA**

| **Groups** | **Mean** | **Standard deviation** | **F value** | **p-value** |
| --- | --- | --- | --- | --- |
| Healthy | 17.0438 | 9.31102 | 3.626 | 0.018* |
| P+NS | 18.0273 | 5.65904 |  |  |
| P+S | 22.7819 | 5.87365 |  |  |
| P+RS | 23.4388 | 6.73925 |  |  |

^*^Statistically significant
