# Supplementary material for: Combined assessment of lysine and N-acetyl cadaverine levels assist as a potential biomarker of the smoker periodontitis
Source: Amino Acids. 2024 Jun 8;56(1):41. doi: 10.1007/s00726-024-03396-4 (PMC11162398; doi:10.1007/s00726-024-03396-4)
Supplement: Supplementary file 12 — Supplementary file12 (DOCX 15 KB) [file 726_2024_3396_MOESM12_ESM.docx]

### **Table S3: Multinomial logistic regression analysis of Polyamines between the groups with healthy subjects as the reference category**

| **Groups** | **B** | **Std. Error** | **Wald** | **df** | **p-value** | **Exp(B)** | **95% Confidence Interval for Exp(B)** | |
| --- | --- | --- | --- | --- | --- | --- | --- | --- |
|  |  |  |  |  |  |  | **Lower Bound** | **Upper Bound** |
| P+NS | 0.018 | 0.047 | 0.153 | 1 | 0.695 | 1.019 | 0.929 | 1.117 |
| P+S | 0.122 | 0.055 | 4.957 | 1 | 0.026 | 1.130 | 1.015 | 1.258 |
| P+RS | 0.139 | 0.056 | 6.030 | 1 | 0.014 | 1.149 | 1.028 | 1.283 |
