# Supplementary material for: Combined assessment of lysine and N-acetyl cadaverine levels assist as a potential biomarker of the smoker periodontitis
Source: Amino Acids. 2024 Jun 8;56(1):41. doi: 10.1007/s00726-024-03396-4 (PMC11162398; doi:10.1007/s00726-024-03396-4)
Supplement: Supplementary file 13 — Supplementary file13 (DOCX 15 KB) [file 726_2024_3396_MOESM13_ESM.docx]

**TABLE S4: Receiver operating characteristic (ROC) curve for screening ability of polyamine.**

|  | **Cut off** | **Sensitivity** | **1-specificity** | **Area under curve** | **Std. Error** | **p-value** | **95% Confidence Interval** | |
| --- | --- | --- | --- | --- | --- | --- | --- | --- |
|  |  |  |  |  |  |  | **Lower Bound** | **Upper Bound** |
| H v/s P+NS | 20.6889 | 0.529 | 0.235 | 0.512 | 0.105 | 0.904 | 0.306 | 0.718 |
| Hv/s P+S | 22.1419 | 0.353 | 0.529 | 0.332 | 0.094 | 0.095 | 0.149 | 0.515 |
| H v/s P+RS | 22.1419 | 0.353 | 0.588 | 0.325 | 0.093 | 0.082 | 0.143 | 0.507 |
| Hv/s P+S | 20.0853 | 0.294 | 0.588 | 0.287 | 0.088 | 0.034 | 0.115 | 0.460 |
| P+NS v/s P+RS | 20.0853 | 0.294 | 0.706 | 0.270 | 0.088 | .022 | 0.098 | 0.442 |
| P+S v/s P+RS | 22.7726 | 0.471 | 0.588 | 0.478 | 0.102 | .823 | 0.278 | 0.677 |
