# Supplementary material for: Combined assessment of lysine and N-acetyl cadaverine levels assist as a potential biomarker of the smoker periodontitis
Source: Amino Acids. 2024 Jun 8;56(1):41. doi: 10.1007/s00726-024-03396-4 (PMC11162398; doi:10.1007/s00726-024-03396-4)
Supplement: Supplementary file 14 — Supplementary file14 (DOCX 17 KB) [file 726_2024_3396_MOESM14_ESM.docx]

**Table S5**. Densitometric analysis of PA bands 1,2,3

| **Table S5A. Band 1** | | | | | |
| --- | --- | --- | --- | --- | --- |
|  | **Groups** | **Mean** | **Standard deviation** | **F value** | **p-value** |
| 1 | Healthy | 1294069 | 796961.2 | \| 4.463926 \| \| --- \| | \| 0.025172 \| \| --- \| |
| 2 | P+NS | 3684165 | 1163373 |  |  |
| 3 | P+S | 1464173 | 855635.5 |  |  |
| 4 | P+RS | 2287761 | 1918263 |  |  |

| **Table S5B. Band 2** | | | | | |
| --- | --- | --- | --- | --- | --- |
|  | **Groups** | **Mean** | **Standard deviation** | **F value** | **p-value** |
| 1 | Healthy | 460925.5 | 49259.75 | 4.83 | 0.00** |
| 2 | P+NS | 3380687 | 441736.2 |  |  |
| 3 | P+S | 1910617 | 368744.1 |  |  |
| 4 | P+RS | 1405591 | 175446.8 |  |  |

^*^Statistically significant

| **Table S5C. Band 3** | | | | | |
| --- | --- | --- | --- | --- | --- |
|  | **Groups** | **Mean** | **Standard deviation** | **F value** | **p-value** |
| 1 | Healthy | 96951.83 | 4987.584 | 9.53 | 0.005** |
| 2 | P+NS | 255315.3 | 119728.4 |  |  |
| 3 | P+S | 966527 | 368527.3 |  |  |
| 4 | P+RS | 751679.8 | 246115.3 |  |  |

^**^Statistically high significant
