# Supplementary material for: Combined assessment of lysine and N-acetyl cadaverine levels assist as a potential biomarker of the smoker periodontitis
Source: Amino Acids. 2024 Jun 8;56(1):41. doi: 10.1007/s00726-024-03396-4 (PMC11162398; doi:10.1007/s00726-024-03396-4)
Supplement: Supplementary file 15 — Supplementary file15 (DOCX 17 KB) [file 726_2024_3396_MOESM15_ESM.docx]

**Table S6**. Pair-wise comparison of PA band intensity.

| **Table S6A. Band 1** | | | |
| --- | --- | --- | --- |
| **S.No.** | **Groups** | ***p-*value** | **Significance** |
| 1 | Total | 0.0251 | * |
| 2 | H vs P+NS | 0.0025 | ** |
| 3 | H vs P+S | 0.8134 | N.S. |
| 4 | H vs P+RS | 0.4539 | N.S. |
| 5 | P+NS vs P+S | 0.0562 | N.S. |
| 6 | P+NS vs P+RS | 0.3416 | N.S. |
| 7 | P+S vs P+RS | 0.5343 | N.S. |

| **Table S6B. Band 2** | | | |
| --- | --- | --- | --- |
| **S.No.** | **Groups** | ***p-*value** | **Significance** |
| 1 | Total | 0.000017 | *** |
| 2 | H vs P+NS | 0.0003 | ** |
| 3 | H vs P+S | 0.0025 | ** |
| 4 | H vs P+RS | 0.0008 | ** |
| 5 | P+NS vs P+S | 0.0114 | # |
| 6 | P+NS vs P+RS | 0.0019 | ## |
| 7 | P+S vs P+RS | 0.0988 | N.S. |

| **Table S6C. Band 3** | | | |
| --- | --- | --- | --- |
| **S.No.** | **Groups** | ***p-*value** | **Significance** |
| 1 | Total | 0.0051 | ** |
| 2 | H vs P+NS | 0.0839 | N.S. |
| 3 | H vs P+S | 0.0150 | * |
| 4 | H vs P+RS | 0.0099 | ** |
| 5 | P+NS vs P+S | 0.0335 | # |
| 6 | P+NS vs P+RS | 0.0348 | # |
| 7 | P+S vs P+RS | 0.4483 | N.S. |
| / |  |  |  |

*indicates significant p value (p<0.05), **indicates significant p value (p<0.01),*** indicates significant p value (p<0.001)
