# Supplementary material for: Combined assessment of lysine and N-acetyl cadaverine levels assist as a potential biomarker of the smoker periodontitis
Source: Amino Acids. 2024 Jun 8;56(1):41. doi: 10.1007/s00726-024-03396-4 (PMC11162398; doi:10.1007/s00726-024-03396-4)
Supplement: Supplementary file 17 — Supplementary file17 (DOCX 15 KB) [file 726_2024_3396_MOESM17_ESM.docx]

**Table S8: Pair-wise comparison of DPPH between the groups using Tukey’s test**

| **(A)** | **(B)** | **Mean Difference (A-B)** | **Std. Error** | **p-value** | **95% Confidence Interval** | |
| --- | --- | --- | --- | --- | --- | --- |
|  |  |  |  |  | **Lower Bound** | **Upper Bound** |
| Healthy | P+NS | 16.36200 | 3.40090 | 0.000** | 7.3568 | 25.3672 |
| Healthy | P+S | 20.27133 | 3.40090 | 0.000** | 11.2661 | 29.2765 |
| Healthy | P+RS | 18.33800 | 3.40090 | 0.000** | 9.3328 | 27.3432 |
| P+NS | P+S | 3.90933 | 3.40090 | 0.661 | -5.0959 | 12.9145 |
| P+NS | P+RS | 1.97600 | 3.40090 | 0.937 | -7.0292 | 10.9812 |
| P+S | P+RS | -1.93333 | 3.40090 | 0.941 | -10.9385 | 7.0719 |

**Statistically high significant, Tukey’s Test
