# Supplementary material for: Combined assessment of lysine and N-acetyl cadaverine levels assist as a potential biomarker of the smoker periodontitis
Source: Amino Acids. 2024 Jun 8;56(1):41. doi: 10.1007/s00726-024-03396-4 (PMC11162398; doi:10.1007/s00726-024-03396-4)
Supplement: Supplementary file 18 — Supplementary file18 (DOCX 14 KB) [file 726_2024_3396_MOESM18_ESM.docx]

**Table S9: Comparison of SOD among the groups using one-way ANOVA**

| **Groups** | **Mean** | **Standard deviation** | **F value** | **P value** |
| --- | --- | --- | --- | --- |
| Healthy | 51.5119 | 5.803214 | 30.86 | 0.000* |
| P+NS | 45.81545 | 10.93263 |  |  |
| P+S | 14.20762 | 15.22263 |  |  |
| P+RS | 15.27507 | 16.44709 |  |  |
