# Supplementary material for: Combined assessment of lysine and N-acetyl cadaverine levels assist as a potential biomarker of the smoker periodontitis
Source: Amino Acids. 2024 Jun 8;56(1):41. doi: 10.1007/s00726-024-03396-4 (PMC11162398; doi:10.1007/s00726-024-03396-4)
Supplement: Supplementary file 19 — Supplementary file19 (DOCX 16 KB) [file 726_2024_3396_MOESM19_ESM.docx]

**TABLE S****10: Receiver operating characteristic (ROC) curve for screening ability of DPPH**

|  | Cut off | Sensitivity | 1-specificity | Area under curve | Std. Error | P value | 95% Confidence Interval | |
| --- | --- | --- | --- | --- | --- | --- | --- | --- |
|  |  |  |  |  |  |  | Lower Bound | Upper Bound |
| H v/s P+NS | 40.2800 | 0.933 | 0.133 | 0.964 | 0.028 | 0.000* | 0.909 | 1.000 |
| Hv/s P+S | 39.5900 | 0.933 | 0.067 | 0.978 | 0.022 | 0.000* | 0.935 | 1.000 |
| H v/s P+RS | 39.4350 | 0.933 | 0.267 | 0.862 | 0.073 | 0.001 | 0.718 | 1.000 |
| Hv/s P+S | 30.1250 | 0.667 | 0.400 | 0.689 | 0.099 | 0.078 | 0.495 | 0.883 |
| P+NS v/s P+RS | 32.5800 | 0.533 | 0.467 | 0.556 | 0.110 | 0.604 | 0.340 | 0.7710 |
| P+S v/s P+RS | 29.7950 | 0.467 | 0.533 | 0.427 | 0.109 | 0.494 | 0.212 | 0.641 |

*Statistically significant, Tukey’s Test
